# Are we giving too much weight to lean mass loss?

**DOI:** 10.1016/j.molmet.2025.102253

**Published:** 2025-09-17

**Authors:** Jeffery Bolte, Annie A. Smelter, Luke Norton

**Affiliations:** 1 Diabetes Division, Department of Medicine, University of Texas Health San Antonio, San Antonio, TX, USA; 2 Department of Cell Systems and Anatomy, University of Texas Health San Antonio, San Antonio, TX, USA

**Keywords:** Obesity, Body composition, Muscle, GLP-1 receptor agonist, Sarcopenia, Aging

## Abstract

The global rise in obesity has underscored the critical importance of body composition, particularly the balance between fat mass and lean mass, in determining health outcomes. While excess fat mass is a well-established risk factor for numerous chronic diseases and reduced longevity, lean mass preservation has been widely considered essential for mitigating fall risk and maintaining functional independence. Recent advances in incretin-based weight loss therapies have shown remarkable efficacy in reducing body weight but have raised concerns about the concomitant loss of lean mass. However, emerging evidence suggests that muscle quality – rather than absolute muscle mass – is a more robust predictor of functional capacity and all-cause mortality. Intriguingly, these therapies may enhance muscle quality even while promoting lean mass loss, offering a nuanced perspective on their impact. This review aims to synthesize current evidence on body composition, muscle quality, and weight loss therapies to guide clinicians in tailoring weight loss strategies that optimize both metabolic health and patient outcomes.

## 1 Introduction

Obesity has become a global health crisis. Obesity and its complications cause morbidity and mortality due to chronic disease, placing significant financial strain on healthcare systems worldwide [1]. Excess adiposity is strongly associated with increased risk of metabolic disorders [2], cardiovascular disease [3], certain cancers [4], and overall reduced life expectancy [5]. Beyond these well-documented risks, the distribution and composition of body mass, specifically the balance between fat mass and lean mass, play a critical role in overall health and functional capacity [6,7].

The treatment for obesity has historically relied on behavioral and lifestyle interventions, most notably diet and exercise. However, recent advancements in therapeutic peptide synthesis have led to the development of new incretin-based medications that promote substantial weight loss in patients with obesity and T2D. The beneficial impact of these new drugs on patients’ metabolic health and quality of life is incontrovertible, however concerns about the potentially negative consequences of lean mass loss persist. Body composition refers to the proportion of total body mass of a particular compartment (fat or lean). Lean mass comprises muscle, bone, organ, connective tissue, and body water (see Table 1 for key definitions). Within the weight loss literature, much effort has been dedicated to optimizing muscle mass preservation. However, many of the methods used to measure muscle mass preservation are poor measures of muscle mass, instead measuring lean mass (see Table 2 for methodology assessing body composition).

**Table 1:** Key terms and definitions.

Term	Definition
Fat Mass	The total mass of fat of the body. Please see Table 2 for measurement methodology [8].
Lean Mass	The non-fat component of the body, which includes muscle mass, bone mass, connective tissue, body organs, water and other materials. Sometimes used interchangeably with “fat-free mass”. Some definitions in the literature exclude bones from this definition, but for the purpose of this review we will consider bones as part of lean mass due to their integral role in the musculoskeletal system and their responsiveness to weight changes [8,9].
Muscle Mass	The total mass of skeletal muscle tissue in the body. Most imaging studies do not provide specific measures of muscle mass independent of total lean mass, but muscle mass is most specifically measured by MRI-based techniques. In other contexts, muscle mass can include smooth and cardiac muscle, but for the purpose of this review we use the term to specifically refer to skeletal muscle mass [8].
Functional capacity	An individual's ability to perform activities that involve physical exertion [10].
Myosteatosis	The infiltration of fat into skeletal muscle [11].
Muscle specific force	Force generation standardized to muscle size. Various methods can be employed to measure and calculate this value from single fiber measurements to whole muscle strength measurements relative to imaging-based muscle cross sectional area [8].
Muscle quality	A broad descriptor of the qualities of muscle. Can refer to histology, imaging, metabolism, or functional assessments. With respect to functional capacity research, it is an ambiguous term which is often used interchangeably in the literature with either muscle specific force or muscle density. Muscle density generally correlates with muscle specific force, an indicator of increased functional performance of muscle relative to its size or mass, and is often assessed as strength per unit muscle mass [8,12]
Muscle density	Radiodensity or attenuation from CT through muscle tissue expressed in hounsfield units (HU). Higher muscle density has been interpreted as muscle tissue with lower fat content [8].
Bone density	The average mineral content of bone. Usually measured by radioattenuation of bone during a DEXA scan, but more precise measures are possible with CT scan and other techniques [13].
Appendicular lean Mass	Proxy for muscle mass in the limbs, usually measured using DEXA-derived non-fat non-bone mass in the four extremities [14].
Sarcopenia (SDOC)	Sarcopenia definitions and outcomes consortium (SDOC) identified grip strength, either absolute or scaled to measures of body size, as an important discriminator of slowness. Both low grip strength and low usual gait speed independently predicted falls, self-reported mobility limitation, hip fractures, and mortality in community-dwelling older adults. Lean mass measured by DXA was not associated with incident adverse health-related outcomes in community-dwelling older adults with or without adjustment for body size [15].

**Table 2:** Methods for Measuring Body Composition.

Method	Description	Strengths	Weaknesses
Bioimpedance	Measures resistive index of electrical conduction, which is a proxy for water and electrolyte content in tissues. Additionally, this method evaluates phase angle to describe hydration status and cell mass [16].	Fast, safe, and easy to use. Phase angle is weakly correlated to mortality.	Assumes fixed hydration for calculation of body composition and output is very sensitive to hydration status, decreasing accuracy.
Anthropometrics	Measures general external proportions including height, weight, head circumference, body mass index, and body circumference using scales, skinfold calipers, and tape measures to assess for adiposity [17].	Fast, accurate for clinical predictions at the population level, inexpensive, and accessible.	Low sensitivity and specificity, inaccurate measurements at the extremes of body mass index and body composition.
Air displacement plethysmography	Measures total body volume via amount of displaced air in a sealed chamber [16].	Straightforward interpretation of results.	Not well tolerated in children, degree of accuracy is not agreed upon in the literature. Difficult to ensure ideal test conditions; results subject to confounding by hydration status, room air flow, and body hair.
Dual-energy X-ray absorptiometry (DEXA)	Whole body X-ray emitting two distinct photon energy levels. Attenuations from these photons are detected and used to determine tissue density [16].	Fast, safe, and easy to use. Low radiation exposure.	Requires a trained radiology technician or physician to administer. Readouts are biased by muscle glycogen and creatine concentrations.
Magnetic resonance imaging (MRI)	Uses differential relaxation properties of hydrogen nuclei in tissues to generate 3D images for analysis of signal intensities in fat and lean tissues [16,18].	Gold standard method for body composition. Accurate at tissue specific level.	Lengthy procedure, very expensive, requires technicians and specialized interpretation.

In this article, we first review the pathophysiology associated with suboptimal body composition and body mass, before discussing the impact of a variety of weight loss methods on lean mass, with a specific focus on patient outcomes. In line with recent trends in pharmaceutical development, we also elaborate on existing and emerging strategies for lean mass preservation and consider whether these approaches should be a priority in the treatment of overweight and obesity. We conclude by discussing which patient populations may benefit from strategies aimed at preserving lean mass in the context of bodyweight reductions.

## 2 Body composition as an indicator of disease risk and metabolic dysfunction

### 2.1 Pathophysiology of excess fat

Insulin resistance is a hallmark feature of obesity and is a major contributor to the cascade of events that ultimately leads to type 2 diabetes (T2D) in genetically predisposed individuals [19]. Insulin resistance is defined as a decreased glucose infusion rate required to maintain normoglycemia during a euglycemic insulin clamp [20]. Free fatty acids, as well as toxic lipid intermediates such as ceramides and diacylglycerols (DAGs), are thought to be the primary mediators of lipotoxicity-induced insulin resistance [[21], [22], [23]]. Weight loss in overweight and obese patients markedly improves measures of insulin resistance, primarily by addressing the underlying mechanisms of lipotoxicity and inflammation [24].

The ectopic deposition of lipid and toxic lipid metabolites in lean tissues incites inflammatory processes and disrupts cellular structure and metabolic function in a tissue-specific manner [22,25]. For example, steatosis in skeletal muscle decreases myocyte contractile force and impairs insulin-stimulated glucose uptake and disposal [25]. In the liver, steatotic hepatocytes demonstrate impaired glucose homeostasis, lipoprotein metabolism, and production of key plasma proteins such as clotting factors and albumin [25,26]. In the cardiovascular system, dyslipidemia promotes atherosclerotic plaque formation in a variety of blood vessels [27], whilst glucotoxicity promotes endothelial damage and microvascular complications [28]. In the pancreas, it is thought that lipotoxicity negatively impacts β-cell function secondary to increased inflammation [22]. Finally, in the brain, dyslipidemia causes glial cells to become dysfunctional, which disturbs the blood brain barrier and may increase the risk for several neurocognitive diseases [29,30]. In patients with obesity, these tissue-specific pathophysiological processes may combine to promote systemic diseases such as T2D, metabolic dysfunction-associated steatotic liver disease (MASLD), cardiovascular disease (CVD), and chronic kidney disease (CKD). Illustrative of this, obese patients with the highest body mass index (BMI) and markers of insulin resistance have a ∼2-fold and ∼3.5-fold increased risk of all-cause mortality, respectively, compared to patients with the lowest values [31].

### 2.2 The metabolic importance of skeletal muscle

Skeletal muscle is an important contributor to a healthy metabolism [32,33], but what defines healthy skeletal muscle may differ depending on the specific context. For the purpose of this review, we use the term “healthy” broadly and intend for this to refer to the skeletal muscle that confers the greatest protection from disease and death while still allowing a person to maintain quality of life.

Muscle is a metabolically active tissue and plays a significant role in whole-body glucose disposal, accounting for approximately 80% of postprandial glucose uptake [34]. In healthy individuals, skeletal muscle removes glucose from circulation via both insulin-dependent and insulin-independent mechanisms [35,36]. When muscle mass is sufficient and muscle cells are metabolically efficient, glucose homeostasis is well maintained due to high insulin sensitivity and sufficient non-oxidative glucose storage capacity [34,37]. In contrast, impaired glucose disposal contributes to hyperglycemia and the microvascular complications associated with insulin resistance [38]. These tightly regulated mechanisms are crucial for sustaining ATP production while preventing glucose-induced microvascular damage.

While skeletal muscle is a primary storage site for glucose, it is not as metabolically active per unit mass in comparison to other tissues. The heart expends 440 kcal/kg/day, the brain 240 kcal/kg/day, and liver and small intestines 200 kcal/kg/day, whereas skeletal muscle only expends 13 kcal/kg/day. Despite its relatively low metabolic activity, muscle is a significant contributor to resting metabolic rate (RMR) due to the mass effect and contributes approximately one-fourth of RMR [39]. However, this is highly variable in humans due to differences in body composition and skeletal muscle oxidative capacity [[40], [41], [42], [43]]. RMR plays a significant role in the metabolic health of patients, particularly in relation to energy balance [44]. When an energy surplus persists over time, it leads to dysregulation of body weight, fat, and inflammation, ultimately contributing to the development of the previously discussed chronic diseases [45].

Lastly, healthy muscle is characterized by functional, efficient mitochondria [46]. Mitochondria are the site of oxidative phosphorylation, a process essential for ATP production but also a major source of reactive oxygen species and oxidative stress [47]. Although the precise relationship between mitochondrial health and the development, prevention and/or progression of diabetes remains debated, it is well established that mitochondrial dysfunction increases oxidative stress, leading to damage of intracellular structures [48]. This contributes to key pathophysiological processes involved in the development of chronic diseases [49]. A major driver of mitochondrial dysfunction is lipotoxicity, which promotes cellular damage and metabolic dysfunction particularly in the setting of obesity and insulin resistance [50]. This underscores the central role of mitochondrial efficiency in maintaining metabolically healthy muscle and highlights the importance of weight loss in mitigating lipotoxic stress.

### 2.3 The relative importance of skeletal muscle mass, strength and quality

Lean mass is essential for mobility, metabolic regulation, and resilience to age-related decline in metabolic health and functional independence [51,52]. It is well documented that patients have better prognoses for various metabolic diseases and lower all-cause mortality in association with lower body fat percentage and higher lean mass, with age as an independent modifier of this relationship [[53], [54], [55]]. In addition to providing metabolic benefits and protection from chronic disease, healthy muscle is the primary contributor to safety of the body. Muscle stretch under load causes a biochemical signaling cascade which increases muscle strength via increased contractile myofibrillar protein synthesis [56,57], and increases tension on the bone matrix, which in turn increases bone density [58]. High bone density protects from debilitating fractures which can cause significant morbidity and mortality, especially in elderly populations [59].

While muscle size is generally predictive of muscle strength, the two are not perfectly correlated and the relationship is highly influenced by “muscle quality”. When muscle is dense, has low levels of myosteatosis, and has high muscle specific force (MSF), it is generally deemed to have high muscle quality. This term often used interchangeably with MSF and muscle density. Muscle density is an imaging-based metric of mass per unit volume of muscle, while MSF is a description of the efficiency of myocyte contraction per unit mass. For further discussion of these definitions, see Table 1. When lipids infiltrate muscle tissues in a dysregulated fashion, they interfere with both energy production and the linear contraction of myofibrils [60,61]. Muscle quality and myosteatosis are significant predictors of mortality [62], even when subjects in the extreme BMI range are excluded, suggesting that muscle quality is an independent predictor of mortality regardless of body composition [62]. The aging process clearly contributes to a decline in muscle size and function, resulting in decreased mobility and impaired quality of life in the later decades [63]. However, the relationship between muscle mass and all-cause mortality is nuanced. A prospective study examining the link between muscle mass, muscle strength, and all-cause mortality found that low muscle mass *per se* was not significantly associated with increased mortality risk [64]. However, in this same study, low muscle strength was linked to an approximate 2.5-fold higher risk of all-cause mortality [64]. This finding is consistent across multiple studies [65,66].

However, a thorough review of the literature on this topic reveals subtleties in this relationship. For example, Weeks et al. found that while both muscle size and density were predictive of muscle function and strength, muscle size was a stronger predictor of strength than muscle density [67]. Weeks’ group notes that their study is unique in that it includes a younger cohort compared to most studies examining myosteatosis and muscle strength. They suggest that in older individuals, myosteatosis may have a greater impact on muscle strength, whereas in younger individuals, muscle cross sectional area (MCSA) may play a more significant role in determining strength. Other studies have established age as an effect modifier of the relationship between muscle strength and either muscle size or muscle density, with a consistent theme that in older age, muscle density is the more predictive factor [68,69]. Taken together, this suggests that in younger individuals, muscle fibers may compensate for myosteatosis, but this compensation diminishes with age.

To summarize, muscle strength is an independent predictor of mortality, but the strength of muscle depends on both muscle quality and muscle size, with age influencing which factor is more predictive. In older individuals (aged >70) who are at the highest risk of death from musculoskeletal injury complications, muscle quality is more strongly linked to muscle strength [70,71]. Evidence suggests that in young, well-trained, and active individuals, the dysregulated storage of lipids in muscle is mitigated, but this does not persist into old age [72]. Muscle contractile capacity, and therefore overall functional capacity, declines progressively with aging, inactivity, and myosteatosis, resulting in weakness. This explains, at least in part, why muscle size alone does not predict all-cause mortality – muscle size is most indicative of strength when the muscle tissue is of high quality [73]. This is an important caveat highly relevant to our discussions below focusing on the impact of weight loss on lean mass, and whether interventions that promote skeletal muscle hypertrophy are warranted.

### 2.4 The relationship between lean mass and fall risk

A specific concern with patients undergoing significant weight-loss interventions is the potential increase in fall and fracture risk due to loss of lean mass and reduced muscle function. Musculoskeletal wasting has been associated with higher frequency, morbidity, and mortality of falls [74]. However, emerging evidence suggests that muscle mass alone is not the most important critical predictor of fall-related outcomes. Instead, muscle strength has been shown to be a stronger and more consistent predictor of fall risk and all-cause mortality in older adults [75]. Ultimately, fall risk is shaped by a complex interplay of factors including environmental conditions, mental status, activity level, muscular strength, and, importantly, body composition [76].

Obesity is a known risk factor for falls; however, evidence suggests that individuals with extreme obesity (BMI >40) may be less likely to sustain hip fractures during falls. This protective effect is likely due to both increased bone density in obese patients due to chronic excess weight bearing as well as increased body surface area, helping to distribute force and reduce the impact experienced by bones during a collision [12,77]. On the other hand, increased body weight also amplifies the overall force transmitted through joints, potentially exacerbating joint damage, both acutely and chronically. While the contribution of these factors varies based on the mechanism of injury, obese patients report higher rates of pain and inactivity following falls compared to non-obese patients [78]. Lastly, it is well established that even in the presence of increased bone density and increased body surface area, excess adiposity and the related insulin resistance and immunodeficiency greatly increase the risk of infection and other complications following surgical interventions to repair orthopedic trauma [79,80]. Post-surgical infections and deep vein thromboses remain common and potentially devastating comorbid conditions among patients suffering from obesity who undergo traumatic falls.

In youth, high body fat percentage and poor body composition are associated with lower muscle quality later in life [81]. Interestingly, BMI has been shown to correlate with higher appendicular lean mass, but not with grip strength in older adults [82]. This observation underscores the importance of preserving strength in critical functional areas such as grip – especially when normalized to body mass – which is a powerful protective factor against falls [83]. When older patients stumble, their ability to grip surrounding structures and support their body weight can be the difference between recovery and a fall [75]. Furthermore, increased grip strength likely serves as a proxy for overall functional capacity and engagement in physical activity, which helps prevent atrophy and maintain mobility. Across a lifetime, leanness is consistently associated with higher muscle strength and quality regardless of BMI, especially in important muscle groups for preventing falls [81]. These findings reinforce the complex interplay between body composition, muscle strength, and overall health outcomes.

Fall risk in elderly individuals follows a U-shaped curve when assessed by weight, with both low and high extremes carrying increased risk. For instance, use of incretin-based therapeutics has been cautioned against in patients with low BMI or an anorexic malnourished phenotype due to their potential to exacerbate fall risk in this population [84]. However, these drugs are not clinically indicated for individuals with anorexia nervosa [85].

This raises an important question: do weight loss interventions reduce muscle strength in critical muscle groups to the extent that fall risk increases? In the following section, we discuss the nuances in treatment outcomes on functional capacity, bone density, and metabolism of behaviorally induced caloric deficits, bariatric surgery, and incretin-based drugs.

## 3 The effects of weight loss on lean mass

While there are various methods to induce weight loss, all strategies share a common foundation: establishing a caloric deficit [86]. Naturally, however, caloric deficits not only reduce fat mass, but also lead to reductions in lean mass. Currently utilized interventions for reducing body fat include, but are not limited to, behaviorally induced caloric deficits (i.e. diet and/or exercise), bariatric surgery, incretin-based drugs such as glucagon-like peptide-1 receptor agonists (GLP-1 RA), stimulants, acarbose, orlistat, and some psychiatric medications. All FDA-approved weight loss interventions work by establishing a caloric deficit via decreased intake, decreased absorption, or mild increases in metabolic rate. Depending on the intervention, patients may experience up to a 30% reduction in weight, along with concurrent improvements in body fat percentage [86]. However, some researchers and clinicians have cautioned against the use of pharmacological weight loss interventions due to the risk of lean mass loss, particularly in the absence of concurrent resistance training [[87], [88], [89], [90], [91]], although there is some decrease in lower body muscle mass to be expected with significant weight loss, as less muscle is required to ambulate a lower body weight.

Here we will discuss in greater detail the metabolic and mechanical effects on lean mass of 1) lifestyle and/or behavior induced caloric deficits, 2) bariatric surgery, and 3) GLP-1 RA medications. We chose these three interventions as they are currently the most efficacious and widely used approaches to induce significant bodyweight reduction.

### 3.1 Behavioral interventions and functional capacity

The effects of behaviorally induced caloric deficits on functional capacity have been extensively studied, but the results are inconsistent. A meta-analysis and systematic review of twenty-seven studies reports improved muscle strength relative to BMI, despite reductions in both lean and fat mass, though this may come at the expense of absolute strength [92]. Given the significant relationship between strength and all-cause mortality, some have raised concerns that caloric restriction-induced weight loss may reduce absolute strength and worsen outcomes. However, in both obese men and women, weight loss is associated with improved balance and decreased fall risk [93,94]. In contrast, data in overweight but not obese patients are limited. A meta-analysis found that older adults with overweight BMI had the lowest fall risk compared to underweight, normal weight, or obese groups, though authors noted possible selection bias and BMI misclassification due to age-related height loss [95].

Overall, the relationship between age, BMI, caloric restriction, and functional capacity is complex. Observational data suggest that elderly individuals may have optimal function at slightly higher BMI than younger adults [95,96]. In frail patients with sarcopenic obesity, calorie restriction resulting in weight loss may worsen outcomes [97,98], though causality is difficult to establish. Physiologic aging complicates interpretation, as declines in appetite, strength, and activity are common late in life. A recent comprehensive discussion by Cortes et al. suggests that even with reductions in lean and bone mass, physical performance can improve with behaviorally induced weight loss and be maintained after weight regain [99]. Therefore, caloric restriction should be personalized, factoring in baseline functional capacity, comorbid conditions, life expectancy, and psychiatric factors – often with a higher BMI target in elderly patients than in younger adults [100].

While caloric restriction alone remains controversial in older adults, there is consistent evidence that elderly individuals with BMI in the low to mid 20s and good overall health tend to have higher functional capacity and outcomes [[100], [101], [102], [103], [104]]. Achieving a BMI in this range may improve function, especially when combined with regular exercise; however, it is recommended that all patients, regardless of weight loss method, consistently adhere to exercise protocols [[105], [106], [107], [108]].

While the impact of caloric restriction on functional capacity is difficult to interpret due to conflicting observational data, the value of exercise as an essential component of any caloric deficit is well supported [109]. Two key studies highlight this: Brennan et al. found that small decreases in strength and VO_2_ max seen with caloric deficit alone were fully prevented when exercise was incorporated [109]. Measures of insulin sensitivity improved equally in both groups after adjusting for greater weight loss in the exercise group. Similarly, Villareal et al. demonstrated that diet plus exercise led to greater improvements in physical function than either exercise or diet alone [110]. Notably, Brennan's study lasted 6 months, while Villareal's extended to a year. While Brennan et al. showed that measures of physical function decreased in the diet alone group, the longer-term study by Villareal et al. showed that after a period of weight maintenance, physical performance recovered.

These studies inform us that it would be best to behaviorally induce a caloric deficit by utilizing both exercise and diet. Both studies showed significant improvements in weight and metabolic status. With overwhelming data showing optimal functional capacity and health outcomes in BMI ranges in the low-to mid-20s, it is advisable that for patients with obesity (without contraindications to weight loss as discussed later), weight loss should be achieved even if only by caloric deficit [[100], [101], [102], [103], [104]]. In conclusion, behaviorally induced caloric deficits in obese and overweight populations are prudent methods for improving functional capacity.

### 3.2 Bariatric surgery and functional capacity

Improvements in functional capacity following bariatric surgery have been documented across a range of functional parameters, including relative muscle strength, gait speed, 400-meter walk time, timed get-up-and-go, and VO_2_ max [[111], [112], [113]] (see Table 3 for description of these functional assessments). These benefits are durable, with studies demonstrating sustained improvements up to five years after surgery, indicating long-term enhancement of physical ability and quality of life [125] that persist even in the context of significant lean mass loss [111,126,127]. In contrast to the mixed results observed with calorie restriction alone, the evidence surrounding bariatric surgery is more consistent: functional capacity improves reliably, even when accompanied by reductions in lean mass. While declines in bone density and elevated fall risk following bariatric surgery warrant consideration (as discussed below in section 3.5), the overall functional gains provide strong evidence that bariatric surgery provides a net benefit to physical function.

**Table 3:** Methods for Measuring Functional Performance.

Assessment	Description	Strengths	Weaknesses
***Strength assessments***			
One repetition maximum leg press	The maximum weight that can be lifted in a single repetition with a leg press machine, reflecting lower limb closed chain maximal strength [114].	Measures maximal gross motor output incorporating a variety of muscle groups. Validated predictor of falls, morbidity, and mortality.	Does not measure open-chain strength and makes no measure of balance or mobility. No cardiorespiratory component. Limited by orthopedic injuries.
Grip strength	Total force during a squeezing motion produced by a single hand. Typically assessed with a dynamometer [115].	The hand is often the limiting point for many activities of daily living. Strong correlation to falls, morbidity, and mortality.	Measures a small muscle group, does not measure balance, mobility, or leg strength (which are other major predictors of falls and mortality). No cardiorespiratory component.
Leg extension	The maximal force generated during a dynamic contraction of the quadriceps (allowing motion across the knee joint) [114].	Quadriceps strength is strongly correlated with falls, morbidity, and mortality. Gives maximal dynamic output for a major muscle group.	Does not capture total leg force output, and poorly simulates walking, running, or stumbling.
Isometric leg extension	The maximal force generated during a static contraction of the quadriceps (without allowing motion across the knee joint) [116].	Quadriceps strength is strongly correlated with falls, morbidity, and mortality. Gives maximal isometric output for a major muscle group.	Does not measure strength under muscle lengthening or shortening. Does not simulate any activity of daily living.
***Cardiorespiratory assessments***			
VO_2_ max	The maximal rate of oxygen consumption during intense exercise, expressed in ml/kg/min [117].	The most tightly correlated single physical measure with mortality. Directly measures maximal cardiorespiratory capacity.	Difficult to administer if directly measured. Sensitive to acute changes in exercise frequency.
Gait speed	The normal, comfortable speed at which an individual walks over a defined distance [118].	Measures an individual's usual exertion during activities of daily living.	Does not represent maximal output, so variable results depending on biasing factors.
400-Meter walk time	The time required to walk 400 m at a self-selected pace [119].	Standardized distance and measures an individual's usual exertion during an extended walk for those who have low baseline function.	Does not represent maximal output, so variable results depending on biasing factors.
6-minute walk test	The total distance walked in 6 min at a self-selected pace [120].	Standardized time and measures an individual's usual capacity during an extended walk for those who have low baseline function.	Does not represent maximal output, so variable results depending on biasing factors.
***Combined functional measurements***			
Sit-to-Stand 5x	The time required to rise from a chair and sit back down five times consecutively. Can be employed with or without use of the arms [121].	Indirectly measures strength to body weight ratio in a safe, controlled setting which represents daily activities.	Low sensitivity for changes over time in patients with high baseline function.
Short physical performance battery (SPPB)	A broad functional test which scores patients based on tests of gait speed, chair stands, and balance tests to assess lower extremity function [122].	Measures a wide variety of components of physical capabilities to form an aggregate of overall functional capacity	Uses submaximal efforts and has limited utility in patients with high baseline function.
Four-step climbing time	The time required to climb four steps [123].	Indirectly measures strength to body weight ratio in a safe, controlled setting which represents daily activities.	Low sensitivity for changes over time in patients with high baseline function.
Physical performance test (PPT)	A broad functional test which scores patients across multiple domains of physical function such as walking, stair climbing, and object manipulation [124].	Provides upper and lower extremity measurements. Measures a wide variety of components of physical capabilities to form an aggregate of overall functional capacity.	Uses submaximal efforts and can be biased by inherent practiced improvement in tests.
Timed up-and-go	The time required to rise from a chair, walk a short distance (usually 3 m), turn, walk back to the chair, and sit down [123].	Indirectly measures strength to body weight ratio in a safe, controlled setting which represents daily activities. Involves mobility and balance.	Low sensitivity for changes over time in patients with high baseline function.
Functional status questionnaire	A self-reported survey instrument that assesses an individual's perceived ability to perform physical, psychological, social, and role functions [124].	Measures patient's symptoms and confidence with their functional status.	High susceptibility to bias, better for primary care setting.

### 3.3 GLP-1 RAs and functional capacity

The effects of GLP-1 RAs on functional capacity are well established, even though direct comparisons with other weight loss methods, such as bariatric surgery or behaviorally induced caloric deficits, are limited. A recent study by Sattar et al. demonstrated that tirzepatide improved muscle fatty infiltration despite a reduction in muscle volume, a change associated with improved muscle function, particularly in older adults [128]. Similarly, Volpe et al. found that semaglutide significantly reduced body mass and fat mass while causing minimal declines in muscle strength, resulting in improved strength per unit mass [129]. This mirrors findings from bariatric surgery, where strength per unit BMI and functional capacity improve, even in the presence of lean mass loss [111,126]. An opinion piece by Conte, Hall, and Klein argued that lean mass loss during GLP-1 RA treatment may not be clinically significant in individuals with obesity [130]. Heymsfield et al. quantified that 10–25% of total weight loss is attributable to muscle mass [131]. Importantly, resistance training during weight loss has been shown to mitigate lean mass loss [132,133]. While no study has directly compared the lean mass-preserving effects of resistance training between GLP-1 RA users and non-users undergoing equal weight loss, one study demonstrated that treatment with GLP-1 RA liraglutide combined with resistance training increased lean mass in patients experiencing weight loss [108].

Additional support for the functional benefits of GLP-1 RAs comes from studies showing improvements in physical performance. For example, liraglutide improves performance on the 6-minute walk test, a surrogate for increased exercise tolerance [134]. Improvements in exercise tolerance likely lower the barrier to physical activity, encouraging long-term improvements in muscle strength [134]. However, during periods of active weight loss, GLP-1 RA treatment – like other forms of caloric deficits – may reduce activity energy expenditure and resting metabolic rate (RMR) [135]. With respect to the decrease in activity energy expenditure, this phenomenon is largely attributed to the decreased energetic requirements to ambulate at a smaller body weight. The decrease in RMR is a contested topic in the literature, which is explored further in the metabolic adaptation section below. Importantly, studies show that once weight stabilizes and energy balance is restored, energy expenditure relative to body weight recovers [136].

Although research into the effects of GLP-1 RA treatment on muscle quality is still developing, there are a few available studies from which we can cautiously extrapolate. Kakegawa et al. used magnetic resonance imaging (MRI) to assess myosteatosis during semaglutide treatment and observed a significant decrease in myosteatosis without concurrent reduction in muscle mass [137]. However, the study was limited by its single-arm pilot design, a small sample size of 21 participants, and relatively young cohort (mean age 53), making it difficult to extrapolate to older populations in whom muscle quality has more pronounced impacts on morbidity and mortality. Similarly, Volpe et al.‘s oral semaglutide study included participants with an average age of 54, again limiting generalizability [138] However, a separate Volpe et al. study investigating semaglutide injections included a more representative population (average age 64) and found significant reductions in fat mass and visceral adipose tissue, with only a mild reduction in fat-free mass index “not associated with a loss in muscle strength” [129]. While these studies unfortunately lacked a placebo control – limiting the ability to attribute outcomes solely to pharmacologic treatment – they consistently suggest that GLP-1 RAs improve muscle quality and functional capacity. Participant behavior, such as changes in physical activity, may have influenced results. Nonetheless, preliminary evidence indicates that GLP-1 RA treatment enhances strength per unit BMI and reduces myosteatosis, thereby lowering long-term fall risk. Early-life improvements in body composition achieved through interventions such as GLP-1 RAs may contribute to muscle quality, preserved function, and reduced morbidity with aging.

### 3.4 Behavioral interventions and bone density

It is established that behaviorally induced caloric deficits can lead to reductions in bone mineral density (BMD) [[139], [140], [141]]. In a two-year study, Villareal et al. reported significant BMD reductions at clinically relevant skeletal sites, suggesting this may be a “potential limitation of prolonged [caloric restriction] for extending life span” [140]. A systematic review by Zibellini et al. concluded that diet-induced weight loss in overweight and obese individuals resulted in “a small decrease in total hip BMD, but not lumbar spine BMD” [141]. However, the authors emphasized that “this decrease is small in comparison to known metabolic benefits of losing excess weight.”

Furthermore, patients with BMIs above 30 experience higher rates of post-operative complications and mortality following orthopedic trauma [79]. This reinforces the clinical benefits of behaviorally induced caloric deficits, despite the associated risk of decreased bone density. However, incorporating exercise – particularly resistance training – during periods of caloric restriction has been shown to mitigate bone density loss [[142], [143], [144]]. For patients hoping to reap the metabolic benefits of behaviorally induced weight loss, concurrent implementation of exercise to promote axial loading of the skeleton is strongly advisable.

### 3.5 Bariatric surgery and bone density

Bariatric surgery results in well-documented decreases in BMD [126,145,146]. These changes are thought to result from altered nutrient absorption secondary to anatomical changes to the gastrointestinal tract [147], as well as profound reductions in caloric intake and decreased axial load on the bony matrix following rapid weight loss [126,145,146]. Together, these factors contribute to increased bone turnover and reductions in BMD.

Several types of bariatric procedures are common, including sleeve gastrectomy, Roux-en-Y gastric bypass, and one-anastomosis gastric bypass. Of these methods, sleeve gastrectomy appears to have the most favorable side effect profile regarding bone density, particularly at clinically important sites such as the hip, lumbar spine, and femoral neck [126,145,146]. In addition to BMD reductions, multiple studies have demonstrated an increased incidence of falls and fall-related injuries following bariatric surgery [[148], [149], [150]]. These risks should be carefully considered between physician and patient when selecting the most appropriate surgical approach for weight loss.

### 3.6 GLP-1 RAs and bone density

GLP-1 RAs present a unique case in the context of bone density compared to bariatric surgery and behaviorally induced calorie deficits. While GLP-1 RAs induce weight loss primarily through appetite suppression and subsequent caloric restriction, they do not involve structural changes to the gastrointestinal tract and therefore lack the malabsorptive complications associated with bariatric surgery. Although clinically relevant BMD loss is observed with caloric deficits and bariatric surgery, a meta-analysis by Li et al. demonstrated that GLP-1 RAs have a neutral effect on BMD. In fact, liraglutide may even improve BMD and reduce fracture risk in patients with T2D undergoing weight loss [151]. The authors acknowledged limitations, such as the predominant use of liraglutide and exenatide in the included studies, short treatment durations, and variability of outcomes. Nonetheless, they concluded that GLP-1 RAs do not consistently increase fracture risk but may, in some cases, modestly increase or decrease it. As newer and more portent agents such as semaglutide and tirzepatide were not included in the meta-analysis, the long-term effects of these medications on BMD have yet to be determined.

## 4 Targeting lean mass preservation during weight loss

Nearly all patients can benefit from improving body composition by safely increasing lean mass and reducing fat mass in ways which maximize functional capacity. Healthy muscle and bone are associated with numerous protective effects, including decreased fall risk, lower likelihood of fractures during falls, fewer complications following injury, enhanced insulin sensitivity, reduced systemic inflammation, and overall improved physical function. While it may seem intuitive that weight loss interventions resulting in lean mass loss would diminish these protective benefits, a growing body of evidence suggests otherwise.

To synthesize the evidence presented thus far, we return to the core question: should muscle mass *per se* be prioritized and preserved during weight loss? To explore this, we examined the impact of weight loss on two primary measures of health.

1) Functional capacity, as measured by muscle quality, VO_2_ max, walk speed, relative strength, and fall risk.
2) Insulin sensitivity, as measured by Homeostasis Model Assessment of Insulin Resistance (HOMA2-IR), euglycemic insulin clamps, and other cardiometabolic biomarkers.

Across all interventions discussed in this review, including behavioral, pharmacologic, and surgical approaches, weight loss consistently improves both functional capacity and insulin sensitivity in overweight and obese patients [112,[152], [153], [154]], even in the presence of reductions in lean mass. Ideally, treatment strategies in all patients would optimize lean mass function using lean mass preserving strategies, however it is difficult to justify foregoing weight loss treatment on account of a decrease in lean mass. That said, as we will discuss next, there are several important factors to consider when designing weight loss strategies that protect lean mass. There are also specific clinical scenarios where weight loss is detrimental and associated with significant and pathologic lean mass loss. However, these situations are typically observed in the context of hypocaloric extremes, and/or distinct pathological processes that drive the weight loss.

### 4.1 Optimizing weight loss to preserve lean mass

The specific approach used for body weight reduction can have important implications for lean mass. For example, the rate of weight loss can influence final body composition. Severe caloric restriction may be less favorable than moderate caloric restriction, as larger deficits typically induce faster weight loss but may compromise lean mass retention [[155], [156], [157]]. An optimal approach from a lean mass preservation perspective would involve slow, sustained weight loss. Rapid weight loss is unavoidable with some interventions, such as bariatric surgery, however this treatment remains a cornerstone of weight-loss therapy and is appropriate in cases where other methods have failed, or rapid results are necessary. Behavioral and pharmacological interventions can use gradual caloric reductions or dose adjustments to slow weight loss.

Rapid weight cycling may also worsen body composition and increase metabolic risk factors after weight regain. Some studies have shown that individuals returning to their starting weight following cycles of weight loss and regain often have higher fat mass and lower muscle mass than if they had maintained their initial weight [158,159]. This phenomenon is largely attributed to the relative ease and speed with which body fat can be gained compared to muscle mass. Some have speculated that this imbalance is exacerbated in the context of recent weight loss due to a decrease in energy expenditure as a neural and hormonal response to prolonged caloric deficits [160]. This phenomenon of “metabolic adaptation” warrants further discussion, as it is a contested topic in the scientific literature.

### 4.2 Fighting against metabolic adaptation

Metabolic adaptation has been identified as a potential drawback of weight loss. This theory suggests that weight loss leads to lasting reductions in RMR and accompanying increases in appetite which may predispose patients to weight regain and persistent reductions in physical activity [161,162]. However, as discussed earlier, long-term studies incorporating a weight maintenance phase have shown that RMR reductions are not persistent, especially when resistance training is included during the weight loss period [163,164].

Importantly, RMR is just one component of total daily energy expenditure (TDEE). Other contributors to energy expenditure include non-exercise activity thermogenesis (NEAT), exercise activity thermogenesis (EAT), and the thermic effect of food (TEF) [165]. During caloric restriction, all components of TDEE similarly decrease [166,167]. While reductions in EAT and TEF can be impacted by conscious decisions to exercise and consume high-protein foods, NEAT is largely governed by neuroendocrine mechanisms out of conscious control [168,169]. While these contributors to RMR decrease during weight loss, they similarly recover during periods of weight maintenance at eucaloric states [136].

In summary, metabolic adaptation does occur during periods of caloric deficits; however, evidence suggests that this relative metabolic slowing resolves once a eucaloric state is reinstated and chronic weight maintenance is achieved [164]. A notable exception may be rapid weight cycling, in which individuals do not experience an extended period of weight maintenance. In such cases, a period of caloric deficit is immediately followed by an extended caloric surplus that surpasses TDEE, resulting in disproportionately greater fat mass gain compared to lean mass. Frequent repetition of this cycle may contribute to cumulative lean mass loss over time [170].

### 4.3 Resistance training during weight loss

Loss of muscle mass during weight loss can often be mitigated through resistance training, particularly when combined with adequate protein intake [132]. Resistance training is the most powerful endogenous intervention known to improve body composition irrespective of changes in weight [171,172]. Resistance training involves generating tension skeletal muscle against a load, activating localized biomechanical signaling cascades that stimulate autocrine and paracrine release of IGF-1 [173,174]. This, in turn, promotes amino acid uptake and synthesis of myofibrillar proteins [175]. Beyond increasing contractile muscle tissue, resistance training also enhances neuromuscular recruitment of muscle fibers, further augmenting strength gains [176].

Other factors influencing strength include myosteatosis and nutritional status [[177], [178], [179]]. Resistance training is generally safe in healthy individuals when performed with manageable loads and without preexisting orthopedic injuries [180]. Its health benefits extend beyond skeletal muscle, impacting metabolic and cardiovascular health [181]. Although aerobic exercise provides some resistance, it does not result in comparable increases in muscle size or strength due to lower loading [182]. During weight loss, prioritizing muscle strength through resistance training is optimal. However, even with consistent resistance training during significant weight loss, patients may still experience decreased muscle size and strength [183]. As discussed above, muscle strength, particularly relative to body habitus, is a far stronger predictor of health outcomes than absolute muscle mass, so modest reductions in muscle size are unlikely to result in negative health outcomes.

While resistance training is a uniquely effective method for maximizing health during weight loss, it may not be feasible for all patients. Barriers such as limited access, physical limitations, or orthopedic issues can prevent consistent adherence. Importantly, patients should not be discouraged from pursuing weight loss on account of not partaking in resistance training. As discussed above, significant health benefits from weight loss – especially reductions in obesity-related disease – can still be achieved in the absence of resistance training.

### 4.4 Pharmacological interventions to enhance lean mass

Currently, there are no FDA-approved pharmacological treatments for sarcopenia, although numerous compounds have been investigated [184]. Historically, oxandrolone, an anabolic steroid, held FDA approval for promoting weight gain after extensive surgery, chronic infections, severe trauma, or prolonged corticosteroid use. It was also used to relieve bone pain associated with osteoporosis, but FDA approval was withdrawn as of 2023 [185,186]. Somatropin, a recombinant growth hormone, remains FDA-approved for several conditions, including HIV-associated wasting and congenital short stature [187,188]. However, systemic administration of growth hormone in patients without a deficiency has limited evidence supporting its efficacy for muscle growth [189]. This is because systemic growth hormone primarily affects visceral organs and bone through endocrine mechanisms, whereas muscle-specific growth is primarily mediated through autocrine and paracrine IGF-1 secretion in response to muscle stretch and damage [190,191]. Testosterone and its derivatives have also been explored as potential treatments for muscle-wasting diseases [[192], [193], [194]]. Despite some anabolic potential, none have gained FDA approval for this indication due to significant adverse effects, including increased risk of neoplasms, virilization, premature epiphyseal closure, psychiatric and behavioral side effects, dyslipidemia, hypogonadism, insulin resistance, infertility, hypertension, erythrocytosis, and coagulopathies [195]. Other endocrine modulators under investigation, but not yet approved, for sarcopenia include selective androgen receptor modulators (SARMS) [196,197], growth hormone secretagogues [196], ghrelin agonists [198], and fast skeletal muscle troponin inhibitors [198].

Recent efforts have increasingly focused on non-endocrine mechanisms of muscle anabolism, particularly targeting the myostatin pathway, a negative regulator of muscle growth [199]. Inhibiting this pathway – often through activin receptor blockade or monoclonal antibodies – has shown promise in both preclinical and clinical studies [[200], [201], [202], [203], [204]]. A leading example of pharmacological targeting of the myostatin pathway is bimagrumab, a well-studied activin type II receptor antagonist. Bimagrumab promotes skeletal muscle growth and fat loss, although it also exerts broader neurohormonal effects [205]. In preclinical models, bimagrumab was shown to increase muscle mass, enlarge myotube diameter, and both prevent and reverse glucocorticoid-induced atrophy in mice [206]. Additionally, it significantly increased trabecular bone mass in both healthy and sarcopenic mice, as well as modestly improving cortical bone area in sarcopenic but not healthy mice [207]. When combined with semaglutide, bimagrumab has demonstrated lean mass protection in diet-induced obese mice [208]. The combination led to greater fat mass loss and ∼10% more lean mass preservation compared to semaglutide alone. Muscle fiber cross sectional area was also normalized, and animals exhibited trends toward improved exercise performance – higher VO_2_ max and longer time to exhaustion – though these differences were not statistically significant [208].

In humans, bimagrumab shows a consistent ability to increase lean mass, but does not appear to improve functional capacity. A small clinical study in non-obese older adults (aged 70–85) demonstrated an increase in thigh muscle volume and total lean body mass after a single dose of bimagrumab, while body adiposity decreased [209]. However, this study did not demonstrate an increase in muscle strength. Other trials of activin receptor inhibitors have corroborated this finding, consistently demonstrating increased muscle mass without corresponding to improvements in strength or physical performance [203,[210], [211], [212]]. In a large, multi-site clinical trial of lean community-dwelling older adults (>70 years), bimagrumab treatment increased lean body mass and reduced fat mass, but had no significant effect on 6-minute walk distance, gait speed, or Short Physical Performance Battery (SPPB) scores [213]. These lean mass gains again failed to translate to improved strength. A 2024 meta-analysis of seven randomized controlled trials in patients with sarcopenia confirmed these findings –bimagrumab improved body composition but not physical performance measures such as grip strength, gait speed, and 6-min walk distance, and more [214].

A Phase 2 trial by Heymsfield et al. evaluated the efficacy of bimagrumab in adults with type 2 diabetes and overweight or obesity [215]. Participants experienced a robust decrease in total fat mass (∼20%) with a significant increase in lean mass (∼4%), as well as a modest improvement in HbA_1C_. However, once again, no meaningful improvements were seen in functional parameters such as grip strength. Moreover, the bimagrumab group experienced a 14% dropout rate due to adverse events (compared to 0% in placebo), with higher total numbers of adverse events – primarily diarrhea and muscle spasms – as well as transient elevations in pancreatic and liver enzymes. The clinical significance and mechanisms of these effects remain unclear. Taken together, these findings suggest that activin receptor blockade reliably improves body composition by increasing lean mass and decreasing fat mass. However, gains in muscle mass have not translated into functional improvements – the key determinant of muscle-related health outcomes, as emphasized throughout this review. Ongoing studies, such as the BELIEVE trial, are investigating the combined use of GLP-1 RAs and activin receptor antibodies to maximize body composition improvements during weight loss. Determining whether these combinations produce sustained improvements on functional capacity will be essential for future clinical recommendations.

## 5 Managing body composition and functional capacity in a clinical setting

Sarcopenia is clinically defined as a “generalized loss of skeletal muscle mass and strength with a risk of adverse outcomes such as physical disability, poor quality of life and death” [216]. This definition emphasizes functional outcomes but leaves much interpretation to the clinician and patient for determining the health of a patient's muscle. According to the European Working Group on Sarcopenia (EWGSOP), sarcopenia is diagnosed by the presence of both low muscle mass and low muscle function (strength or performance), tying its relevance to a patient's lifestyle and physical demands [216]. The interventions discussed in this review – behavioral, surgical, and pharmacologic – are all associated with improvements in functional capacity and quality of life in patients with overweight or obesity. As such, these interventions may be important clinical tools to mitigate sarcopenic phenotypes, despite often involving some degree of lean mass loss.

The Sarcopenia Definition and Outcomes Consortium reinforces this view. It “identified grip strength, either absolute or scaled to measures of body size, as an important discriminator of slowness. Both low grip strength and low usual gait speed independently predicted falls, self-reported mobility limitation, hip fractures, and mortality in community-dwelling older adults. Lean mass measured by DXA was not associated with incident adverse health-related outcomes in community-dwelling older adults with or without adjustment for body size” [15]. This commentary highlights important distinctions between the sarcopenic phenotype and the phenotype of obese patients undergoing treatment-induced weight loss. Nearly all clinical markers of sarcopenia trend in the opposite direction during treatment-induced weight loss. For example, GLP-1 RA use may reduce absolute grip strength, but grip strength relative to BMI improves. Gait speed (e.g., 6-minute walk test) improves with treatment induced weight loss but is reduced in sarcopenia. Risk of morbidity and mortality following falls and fractures declines with weight loss (except in certain bariatric surgeries), while they rise in sarcopenia. Mortality decreases after weight loss but increases with sarcopenia. These findings challenge concerns about lean mass loss for patients undergoing treatment-induced weight loss. Therefore, when weight loss is clinically indicated in obese patients, interventions to preserve lean mass are not necessary to achieve improvements in functional capacity, mortality, and morbidity.

It is likely that among patients above the normal BMI range, and without the comorbidities discussed in the next section, some form of weight-reducing intervention that decreases adiposity and improves functional capacity will be beneficial, regardless of concurrent efforts to maintain lean mass. Data consistently show improved functional capacity at lower body fat percentages and BMI ranging between 18.5 and 25, with the caveat that “normal” BMI may be slightly higher in older adults due to predictable age-related changes in stature and body habitus. To date, there is no compelling evidence that any of the weight loss approaches discussed in this review consistently result in adverse health outcomes due to decreased lean mass.

It is crucial to reiterate that in the presence of increasing myosteatosis with old age and inactivity, patients may improve their functional status simply by reducing their weight, thereby increasing relative strength and muscle quality [73]. With few exceptions, efforts to preserve lean mass – which may require polypharmacy, greater medical expenses, and increased side effect profiles – are not necessary to preserve or even increase functional capacity. Aging and inactivity cause changes in muscle fiber type, contractility, and lipid infiltration, all of which impair function. As discussed earlier, in young adults, muscle size is the best predictor of muscle strength, but in older adults, muscle quality becomes a more accurate measure of strength. In both groups, lower adiposity is associated with improved functional capacity. Medical interventions that reduce excess adiposity remain central to interrupting this positive feedback loop and improving long-term function, regardless of concurrent lean mass preservation.

Studies have even shown that in patients with overweight (25 < BMI<29.9) or class I obesity (30 < BMI<34.9), improvements in muscle quality are predictive of better functional outcomes – perhaps more so than the absolute change in weight [73,103,217,218]. This suggests that even patients who are overweight, but not obese, can benefit from weight loss when excess adiposity and myosteatosis are present. This contradicts the prevailing concern that weight loss may worsen sarcopenia. GLP-1 RAs are the most powerful, consistent, and well-tolerated method of weight loss available. They reduce muscle fat infiltration and improve strength-to-BMI ratios. As such, they should not be avoided in overweight older patients at risk for sarcopenia, especially those with impaired glucose metabolism.

### 5.1 When is lean mass preservation a clinical priority?

Despite the clear distinction between sarcopenic lean mass loss and treatment-induced weight loss, there are clinical situations in which preserving lean mass during treatment-induced weight loss becomes prudent. Patients most likely to benefit from lean mass-preserving strategies are those who already exhibit clinical signs and symptoms of significantly impaired muscle function impacting their health and autonomy, regardless of excess body mass. One such group includes individuals with both significant overweight/obesity and substantial muscular atrophy due to extended periods of immobility or inactivity. This can occur in post-surgical patients, those with neurologic deficits, patients immobilized by obesity-related disability, or those recovering from major injuries. For these patients, pharmacologic interventions to preserve muscle mass may be warranted before pursuing further interventions such as physical therapy, surgery, or significant weight loss.

Another group which may stand to benefit includes patients experiencing cachexia – a pathologic form of lean mass loss often associated with chronic illness and often accompanied by weight loss. Cachexia is defined as a complex metabolic syndrome involving muscle loss, with or without fat loss, and differs mechanistically and clinically from treatment-induced weight loss [219]. Unlike intentional weight loss in obesity, cachexia is associated with profound insulin resistance and decreased functional capacity [220,221].

Patients with concurrent conditions known to worsen with low muscle mass may also be candidates for pharmacologic lean mass-preserving strategies during weight loss. These diseases include certain forms of cancer [222,223], cirrhosis [224], respiratory disease [225], and certain cardiovascular diseases [225].

Patients with advanced diabetes who are on the low range of clinical overweight/obesity may also benefit from lean mass preservation. These individuals often have poor baseline muscle quality and mass, and diabetic nephropathy can lead to significant protein loss in the urine, decreasing substrate availability for muscle maintenance [226,227]. Similarly, obese patients with prior musculoskeletal injury leading to significant muscle atrophy could benefit from interventions that preserve lean mass during weight loss.

Evidence strongly supports the link between increased muscle strength and reduced all-cause mortality [228,229]. Although resistance training is the most effective and broadly beneficial intervention for maintaining or increasing lean mass, many patients face significant barriers to this strategy. While pharmacologic interventions to improve muscle mass and strength have shown mixed efficacy and notable side-effect profiles, their potential benefits justify ongoing research. Identifying specific clinical contexts in which the benefits of pharmacological lean mass preservation outweigh the risks remains an important goal. For patients unable to engage in exercise, exploring safe and effective alternatives for achieving a strong phenotype is a valuable therapeutic objective.

Low BMI is associated with worse prognoses in various pathologies including peripheral artery disease [230,231], hemorrhagic stroke and subarachnoid hemorrhage [232], pelvic and acetabular fractures [233], sepsis [234], heart failure [235], and several cancers [[236], [237], [238], [239], [240]]. These conditions often exhibit a U-shaped relationship between BMI and mortality, with the lowest mortality observed at a healthy BMI (18.5–25), and significantly increased mortality at BMIs below 18.5 or above 30. A similar U-shaped relationship exists for morbidity and mortality when scaled to body fat percentage. However, having a low body fat percentage without low BMI does not correlate with poor outcomes [45,[241], [242], [243], [244]]. Padwal et al. found that the highest all-cause mortality in both men and women was observed in individuals with extremely low BMI coupled with high body fat percentage, a description consistent with a cachexic phenotype.

Low muscle mass, independent of fat mass or BMI, is associated with poor outcomes in diseases such as many cancers [222,223], cirrhosis [224], respiratory disease [225], and some cardiovascular diseases [225]. For certain conditions, such as pancreatic cancer, muscle mass loss is a stronger predictor of outcomes than fat mass loss, regardless of BMI [245,246]. Muscle mass appears to play a protective role, whether through metabolic or mechanical mechanisms. Interventions that reduce muscle size or function could be life-threatening in advanced pathologies where low muscle mass is linked to increased mortality.

While the outcomes of patients with these diseases follow a U-shaped curve with respect to BMI and mortality, the incidence of many chronic diseases is inversely correlated with BMI [247]. Patients with low BMI and a muscular body composition have the lowest incidence of chronic disease. As such, the decision to pursue weight-loss treatment must consider the specific clinical context, balancing the potential benefits against the risks posed by preexisting conditions. For instance, a patient with no disease or subclinical early-stage disease and high BMI may benefit from weight-loss interventions, while a patient with late-stage pathology and moderately elevated BMI may not. Patients with normal BMI who have conditions associated with increased mortality at lower weights could also be harmed by weight loss. Similarly, body fat percentage, regardless of BMI, is an independent predictor of the appropriateness of body composition interventions. For example, a patient with normal BMI but high body fat percentage and T2D may benefit from mild weight-loss treatment aimed at body fat reduction and improved glucose tolerance to prevent incidence and progression of disease. Meanwhile, a patient with mildly elevated BMI, high body fat percentage, and advanced cancer, cirrhosis, or severe heart failure may not. Ultimately, the risks and benefits depend on the patient's current conditions and their intrinsic and extrinsic risk factors. Much research on newer forms of weight loss treatment and lean mass preservative treatment is necessary before any evidence-based conclusions can be drawn about the variety of nuanced factors influencing outcomes during weight loss in the context of specific pathologies.

## 6 Conclusion

In summary, this analysis underscores the clinical challenges associated with extremes of weight and body composition. Both underweight and overweight states are linked to increased fall risk, higher mortality from numerous diseases, and diminished quality of life. The decision to pursue weight loss treatment is a nuanced clinical consideration that requires balancing the risks associated with metabolic disease against those of clinical frailty. Notably, the hazard ratios for all-cause mortality associated with severe insulin resistance and low muscle strength are similar (approximately threefold), emphasizing the need for cautious and individualized interventions.

GLP-1 RA medications, while effective for weight loss, have raised concerns about potential long-term frailty risk, such as reductions in bone mineral density and muscle mass. However, unlike acute behavioral caloric restriction, current data on GLP-1 RAs in otherwise healthy individuals have not demonstrated compromised bone integrity or muscle function. In many cases, these medications have been associated with reduced fracture risk and improved muscle function metrics. Continued monitoring and research will be essential to fully understand the long-term safety and efficacy of these increasingly powerful medications. Among weight loss agents, GLP-1 RAs stand out as minimally invasive, highly effective, and generally well-tolerated.

Muscle quality, closely linked to myosteatosis, becomes a crucial predictor of strength as individuals age. Early interventions that reduce myosteatosis can enhance muscle function and prevent age-related functional declines. Chronic obesity increases the risk of various of conditions, and maintaining a normal weight significantly reduces the risk of premature mortality. However, specific scenarios – such as rapid weight cycling, extreme caloric deficits, and weight loss in the context of end-stage chronic diseases – should generally be avoided due to their association with poorer body composition and outcomes. While muscle mass contributes to longevity, muscle strength is a far more robust predictor. Pharmacological interventions aimed at enhancing lean mass often come with significant side effect profiles, and their long-term safety and efficacy remain uncertain. Many of these treatments may increase muscle mass without improving strength, particularly in the absence of a training stimulus. For *optimal* health and longevity, achieving and maintaining a healthy weight should be accompanied by resistance training to maximize muscle mass, strength, and bone mineral density. However, in patients with obesity and no contraindicating comorbidities, evidence-based weight loss strategies should be encouraged without undue concern for lean mass loss.

## CRediT authorship contribution statement

**Jeffery Bolte:** Writing – review & editing, Writing – original draft, Investigation, Conceptualization. **Annie A. Smelter:** Writing – review & editing, Writing – original draft, Investigation, Conceptualization. **Luke Norton:** Writing – review & editing, Project administration, Conceptualization.

## Funding

Supported by 10.13039/100000002NIH 10.13039/100000062NIDDK - R01DK128247 and R01DK129676 (LN).

South Texas Medical Scientist Training Program NIH NIGMS/T32GM113896 and T32GM145432 (AAS).

## Declaration of competing interest

All authors declare no competing personal or financial interests.

## Data Availability

No data was used for the research described in the article.
